# Peroperative Notification of Schwartze Sign in Otosclerosis

**DOI:** 10.1002/oto2.70021

**Published:** 2024-10-06

**Authors:** Kanu Lal Saha, Farhan Tarafder

**Affiliations:** ^1^ Department of Otolaryngology–Head and Neck Surgery Bangabandhu Sheikh Mujib Medical University Dhaka Bangladesh

**Keywords:** active stage, otosclerosis, peroperative, Schwartze sign

A microscopic photograph of a 22‐year‐old male, clinically and audiologically diagnosed with otosclerosis, revealed a normal‐appearing tympanic membrane (Figure 1A). After tympanomeatal flap elevation, a Schwartze sign, characterized by diffuse red swelling over the promontory, was observed but excluded as a tumor‐like lesion, paraganglioma^1^ due to the absence of attachment to Jacobson's nerve and the lack of pulsation (Figure 1B). The malleus and incus were noted to be normal and mobile. However, the stapes footplate was found to be white, thickened, sclerotic, and immobile (Figure 1C). A stapedotomy was performed using a 0.7 mm skeeter drill, followed by the insertion of a 0.6 × 4.5 mm Teflon piston (Figure 1D and E).

**Figure 1:**
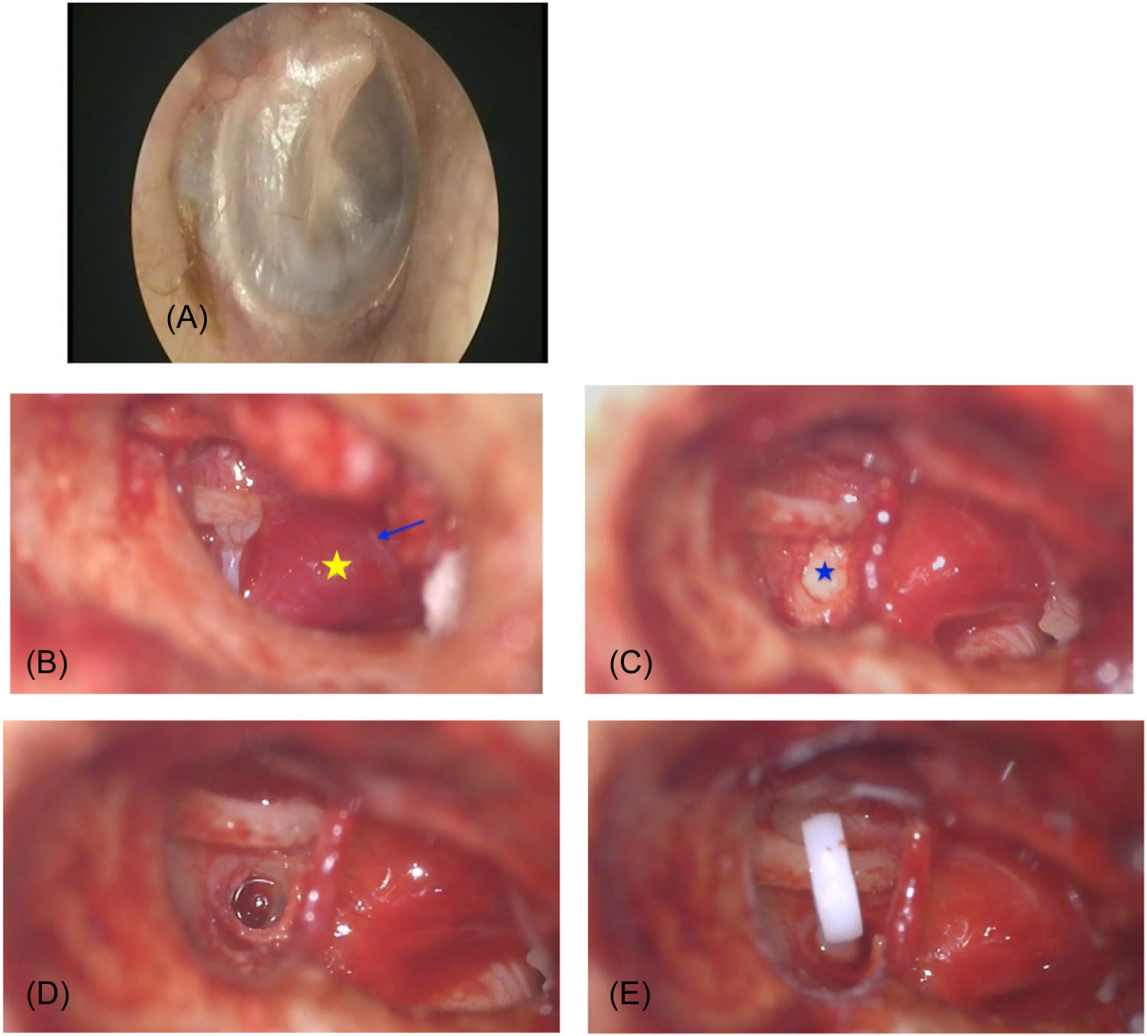
(A) Otoendoscopy showing a normal tympanic membrane. (B) Reddish diffuse area over the promontory (yellow star), indicative of Schwartze sign, with Jacobson's nerve marked (blue arrow). (C) Otosclerotic footplate (blue star). (D) Perforation of the footplate. (E) Placement of the prosthesis between the footplate and the long process of the incus.

Schwartze sign, a characteristic feature of otosclerosis, typically presents as a reddish discoloration over the promontory, visible through an intact tympanic membrane.^2^ In this case, however, the tympanic membrane appeared transparent and shiny, with no discoloration. A diffuse reddish area consistent with Schwartze sign was observed over the promontory after lateral tympanotomy. This sign indicates the proliferation or dilatation of blood vessels within the middle ear, suggesting an active phase of otosclerosis, which may contraindicate surgical intervention.^3^, ^4^

Sodium fluoride therapy can help stabilize the active stage, resulting in a less vascular surgical field and potentially reducing postoperative complications, such as hearing loss.^4^, ^5^ However, in this case, significant improvement in hearing was achieved, with air conduction and bone conduction averaging 17.5 dB and 10 dB, respectively, and an air‐bone gap of 7.5 dB in the operated ear, without the use of preoperative sodium fluoride therapy.

To the best of our knowledge, intraoperative pictorial evidence of Schwartze sign in otosclerosis has not been previously documented.

## Author Contributions

**Kanu Lal Saha**, developed the concept of the work, drafted the manuscript, or revised it critically, approved the final manuscript before submission; **Farhan Tarafder**, drafted the manuscript, approved the final manuscript before submission.

## Disclosures

### Competing interests

The authors declare that there are no conflicts of interest regarding the publication of this article.

### Funding source

This study received no specific grant from any funding agency.
